# Durability of immunogenicity at 5 years after a single dose of human papillomavirus vaccine compared with two doses in Tanzanian girls aged 9–14 years: results of the long-term extension of the DoRIS randomised trial

**DOI:** 10.1016/S2214-109X(24)00477-7

**Published:** 2025-01-29

**Authors:** Deborah Watson-Jones, John Changalucha, Caroline Maxwell, Hilary Whitworth, Paul Mutani, Troy J Kemp, Beatrice Kamala, Jackton Indangasi, George Constantine, Ramadhan Hashim, David Mwanzalima, Rebecca Wiggins, Devis Mmbando, Nicholas Connor, Miquel A Pavon, Brett Lowe, Saidi Kapiga, Philippe Mayaud, Silvia de Sanjosé, Joakim Dillner, Richard J Hayes, Charles J Lacey, Ligia Pinto, Kathy Baisley

**Affiliations:** a Mwanza Intervention Trials Unit, National Institute for Medical Research, Mwanza, Tanzania; b Faculty of Infectious and Tropical Diseases, London School of Hygiene & Tropical Medicine, London, UK; c Faculty of Epidemiology and Population Health, London School of Hygiene & Tropical Medicine, London, UK; d HPV Serology Laboratory, Frederick National Laboratory for Cancer Research, Leidos Biomedical Research, Frederick, MD, USA; e York Biomedical Research Institute & Hull York Medical School, University of York, York, UK; f Infection and Cancer Laboratory, Cancer Epidemiology Research Program, ICO-IDIBELL, Barcelona, Spain; g Centre for Biomedical Research in Epidemiology and Public Health Network (CIBERESP), Madrid, Spain; h Institute of Global Health (ISGlobal), Barcelona, Spain; i National Cancer Institute (NIH), Rockville, MD, USA; j Karolinska Institute, Stockholm, Sweden

## Abstract

**Background:**

WHO has recommended that one dose of human papillomavirus (HPV) vaccine can be given to individuals aged 9–20 years to prevent HPV infection. Estimating durability of immune responses after a single dose in the target age for vaccination is important. We report immunogenicity results in Tanzanian girls up to 5 years after receiving a dose.

**Methods:**

In this open-label, randomised controlled trial (the Dose Reduction Immunobridging and Safety Study of Two HPV Vaccines in Tanzanian Girls [DoRIS] trial), 930 Tanzanian schoolgirls aged 9–14 years were enrolled and randomly allocated to receive one, two, or three doses of either the two-valent vaccine (Cervarix; GSK, Wavre, Belgium) or nine-valent vaccine (Gardasil-9; Merck Sharp & Dohme, Haarlem, Netherlands). Seropositivity specific to HPV16 or HPV18, antibody geometric mean concentrations (GMCs), and antibody avidity were measured annually up to month 36. Participants in the one-dose and two-dose groups were followed annually in a long-term extension of the DoRIS trial to month 60; the primary outcome was seropositivity specific to HPV16 or HPV18 comparing one dose with two doses.

**Findings:**

Single-dose seropositivity for HPV16 IgG antibodies at month 60 with either vaccine was more than 99% and non-inferior to two doses. 98% of girls in the one-dose two-valent vaccine group and 93% in the one-dose nine-valent group were seropositive for HPV18 at month 60; however, the non-inferiority criteria for HPV18 seropositivity comparing one dose with two doses were not met. Although HPV16 and HPV18 antibody GMCs after one dose were lower than those observed after two doses, antibody GMCs in the one-dose groups remained stable from month 12 to month 60. There was no evidence of a difference between the one-dose and two-dose groups in HPV16 or HPV18 antibody avidity at month 36 for either vaccine.

**Interpretation:**

A single dose of HPV vaccine in girls aged 9–14 years continues to provide stable immune responses 5 years after vaccination, although ongoing surveillance for potential waning immunity after a single dose is needed. Participants are being followed up to 9 years after vaccination.

**Funding:**

UK Department of Health and Social Care, UK Foreign, Commonwealth & Development Office, Global Challenges Research Fund, UK Medical Research Council, and the Wellcome Trust through the Joint Global Health Trials Scheme; Bill & Melinda Gates Foundation.

## Introduction

Effective prophylactic vaccines to prevent infection with human papillomavirus (HPV), the primary cause of cervical cancer, have been available since 2006. WHO targets for cervical cancer elimination include 90% of girls being fully vaccinated by the age of 15 years by 2030.^1^ However, in 2022, only 21% of girls in the target age group for HPV vaccine (9–14 years) were estimated to be fully vaccinated with the recommended multidose schedules.^2^ Challenges to HPV vaccine introduction, uptake, and delivery include the costs of vaccinating girls and the capacity to introduce and sustain a multidose vaccination programme.^3^, ^4^

Potential advantages of a single-dose HPV vaccine regimen include reduced costs, ease of delivery, and potentially increased acceptability. Observational studies that initially provided evidence for the efficacy and immunogenicity of one dose came from the Costa Rica Vaccine Trial (CVT), which offered the two-valent vaccine Cervarix (GSK, Wavre, Belgium), and the International Agency for Research on Cancer (IARC) India trial, which offered the four-valent vaccine Gardasil (Merck Sharp & Dohme, Haarlem, Netherlands).^5^, ^6^ In these studies, some participants did not complete their full multidose schedule and were followed up as observational cohorts. Single-dose recipients had high HPV16 or HPV18 seropositivity but lower geometric mean concentrations (GMCs) of HPV16 and HPV18 IgG antibodies compared with those who received two or three doses. However, all doses had similar efficacy against incident or persistent HPV16 or HPV18 infection.^7^, ^8^ This protection was sustained up to 9 years in the IARC India study and 11 years in the CVT.^6^, ^9^

**Research in context**

**Evidence before this study**

The single-dose human papillomavirus (HPV) vaccination schedule has been shown to provide protection against persistent HPV16 and HPV18 infection for up to 11 years, in the context of observational studies of women who did not complete their multidose vaccination schedules in two randomised trials (Costa Rica Vaccine Trial and International Agency for Research on Cancer [IARC] India HPV vaccine trials). The first randomised controlled trial of single-dose HPV vaccine efficacy, conducted in females aged 15–20 years in Kenya (KEN SHE), showed an efficacy against incident persistent HPV16 and HPV18 infection of more than 97% at 36 months. The Dose Reduction Immunobridging and Safety Study of Two HPV Vaccines in Tanzanian Girls (DoRIS) trial in Tanzania, the first randomised trial of the single-dose schedule in girls in the target age range for HPV vaccination (9–14 years), showed that more than 98% of girls who received one dose were seropositive for HPV16 or HPV18 IgG antibodies at 24 months, and had antibody concentrations that were non-inferior to those in one-dose recipients in the KEN SHE trial. WHO approved the off-label use of a single-dose schedule in females and males aged 9–20 years on the basis of these studies. However, data on durability of immune responses in young adolescents (aged <15 years) are insufficient. To search for studies of long-term follow-up after a single dose of HPV vaccine in young girls, we searched PubMed for articles published between Aug 10, 2020 (the date of the last review of the evidence for single dose HPV vaccination, published by the Single-Dose HPV Vaccine Evaluation Consortium) and April 21, 2024, using the terms “human papillomavirus” AND “vaccine” AND (“immunogenicity” OR “efficacy” OR “effectiveness”) AND “single dose” AND “long-term”. This search identified one publication showing sustained antibody concentrations up to 10 years among girls who received a single dose of HPV vaccine at age 10–14 years in the IARC India trial. A study in Fiji of women who were vaccinated through the national HPV vaccination programme at age 9–12 years showed 81% vaccine effectiveness of one dose against prevalent HPV16 or HPV18 infection over 8 years. No other studies of long-term follow-up of the single-dose regimen in individuals who were vaccinated at younger than 15 years were identified.

**Added value of this study**

The immunogenicity results from the DoRIS trial after 5 years of follow-up show that HPV16 seropositivity 5 years after a single dose of HPV vaccine was more than 99%, and similar to that in the two-dose groups. HPV18 seropositivity at 5 years was lower in the single-dose groups than in the two-dose groups, but was still high (>93%). HPV16 and HPV18 antibody concentrations after a single dose reached a plateau at 12 months and remained stable up to 5 years. The antibody trajectories over time after a single dose are similar to those observed in studies of a single dose in older females from different geographical locations in whom efficacy has been shown.

**Implications of all the available evidence**

To our knowledge, this is the first randomised controlled trial of the single-dose regimen to show that a single dose of HPV vaccine in girls aged 9–14 years produces durable antibody responses that remain stable up to 5 years. This is also the first study of long-term immunogenicity of a single dose of nine-valent vaccine. These data, combined with single-dose efficacy data from the KEN SHE trial and the Costa Rica Vaccine Trial and IARC India studies, continue to support the WHO recommendation for a single-dose HPV vaccine regimen.

Data are now available from two randomised trials of single-dose HPV vaccination. The KEN SHE trial, the first randomised trial of single-dose efficacy, enrolled sexually active Kenyan females aged 15–20 years who were randomly allocated to a single dose of either two-valent vaccine (Cervarix), nine-valent vaccine (Gardasil-9), or a control vaccine (meningococcal vaccine).^10^ The trial reported 97·8% efficacy against persistent HPV16 or HPV18 infection for the two-valent and 98·8% for the nine-valent vaccine at 36 months after vaccination.^11^ The Dose Reduction Immunobridging and Safety Study of Two HPV Vaccines in Tanzanian Girls (DoRIS) trial, the first randomised trial of the single-dose regimen in the target age group for HPV vaccination, compared immune responses in Tanzanian girls aged 9–14 years after one, two, or three doses of the same two HPV vaccines as offered in the KEN SHE trial.^12^ At 24 months, more than 99% of girls in the one-dose groups were seropositive for anti-HPV16 antibodies and non-inferiority of HPV16 seropositivity was shown for one dose compared with two or three doses for both vaccines.^13^ More than 98% of girls in the one-dose groups of both vaccines were anti-HPV18 antibody positive at month 24, although the predefined non-inferiority criteria were not met for HPV18 seropositivity. Single-dose antibody responses peaked at month 1 and then fell slightly before plateauing between months 12 and 24. Similar observations have been recorded in the CVT and IARC India studies, where single-dose antibody concentrations plateaued and remained stable up to 11 years.^9^, ^14^ Immunobridging comparisons of antibody GMCs in the one-dose groups in the DoRIS trial showed that HPV16 and HPV18 GMCs and seropositivity for both vaccines were non-inferior to those in the one-dose groups in the KEN SHE, CVT, and IARC India studies, where single-dose efficacy has been shown.^15^, ^16^

The KEN SHE and DoRIS trials contributed to the evidence that led to the recent recommendation by WHO for a one-dose schedule in individuals aged 9–20 years.^17^ However, a limitation of the results was the relatively short follow-up period in young girls. Data on the long-term durability of immune responses when a single dose is given to girls in the target age range for vaccination are needed. These data will provide important evidence of likely ongoing protection with a single-dose regimen over time that could inform policy makers still considering a single-dose programme. In this study, we present the 5-year (month 60) immunogenicity data from the DoRIS trial long-term follow-up, comparing antibody responses after one or two doses of the two-valent or nine-valent vaccines in Tanzanian girls. We also report on antibody avidity, a measure of how strongly an antibody binds to its target antigen**,** at 3 years.

## Methods

### Study design

DoRIS was an open-label, randomised, non-inferiority immunobridging trial in Tanzania comparing the immune responses and safety of one, two, and three doses of the two-valent virus-like particle (VLP) HPV vaccine (Cervarix) and the nine-valent VLP HPV vaccine (Gardasil-9). All participants were followed until month 36. Participants in the one-dose and two-dose groups were invited to join a long-term follow-up extension of the DoRIS trial, where they will be followed up to 9 years (month 108). The three-dose groups were not invited for long-term follow-up because most countries have discontinued provision of three-dose regimens in this age group and there are extensive data on three-dose schedules from earlier clinical trials. The trial and its extension were approved by the ethics committees of the Tanzanian Medical Research Coordinating Committee and the London School of Hygiene & Tropical Medicine, with regulatory approval from the Tanzania Medicines and Medical Devices Authority. This study is registered with ClinicalTrials.gov, NCT02834637.

### Participants

930 girls were enrolled into the main DoRIS trial from primary and secondary schools in Mwanza, Tanzania between Feb 23, 2017, and Jan 6, 2018.^12^, ^13^ Girls were eligible if aged 9–14 years, healthy, HIV negative, planning to reside in Mwanza for 36 months, and willing to give informed assent. Girls were excluded if they had received a prophylactic HPV vaccine; had a history of genital warts, cervical lesions, or past treatment following positive cervical cancer screening; or were pregnant, immunocompromised, or unwell. Written or fingerprinted informed parental or guardian consent and written or fingerprinted assent from potential participants were obtained before screening and vaccination. At the end of the main trial, girls in the one-dose and two-dose groups and their parents or guardians were given information about the extension. Girls younger than 18 years were asked for written or fingerprinted assent, with written or fingerprinted consent from their parent or guardian. Participants aged 18 years or older were asked for written or fingerprinted consent.

### Randomisation and masking

Participants were randomly allocated (1:1:1:1:1:1) to one of six trial groups: three doses given over 6 months, two doses given 6 months apart, or a single dose of either the two-valent or nine-valent vaccine. The randomisation list was computer-generated by an independent statistician, using random permuted block sizes of 12, 18, and 24. Allocation was concealed from the study team and participants using sequentially numbered sealed opaque envelopes. After allocation, participants and clinic staff were made aware of the participant's trial group.

### Procedures

Full details of the trial procedures have been described previously.^12^ In brief, eligibility screening was done within 30 days before randomisation. At screening, girls (or their parent if the girl was aged <12 years) had to pass a test of understanding. Three attempts were allowed before a girl was considered not to have passed screening. At the day 0 enrolment visit, participants were randomly allocated to trial group and blood samples were collected for baseline immunogenicity. Two nurse-assisted, self-administered vaginal swabs were collected for baseline HPV DNA testing and genotyping with the Anyplex II HPV 28 detection assay (Seegene; Seoul, South Korea) at the Catalan Institute of Oncology (Barcelona, Spain).

Participants were vaccinated according to their study group and attended the clinic 1 month after each vaccination visit to record any adverse events. Whole blood samples of approximately 15–20 mL were collected for immunological assays at day 0 and months 1, 7, 12, 24, 36, and 60, and will be collected at months 84 and 108. During the trial extension, to help ensure a high rate of retention, participants are contacted or sent an SMS reminder about the trial every 3 months and visited at home at least annually. Participants in the extension will be offered HIV testing at months 84 and 108.

HPV16 and HPV18 serum IgG concentrations were measured using an L1 VLP ELISA at the HPV Serology Laboratory of the Frederick National Laboratory for Cancer Research (Frederick, MD, USA).^18^ Antibody seropositivity was defined as a concentration equal to or greater than the assay threshold: 1·309 IU/mL for HPV16 and 1·109 IU/mL for HPV18. The HPV16-specific and HPV18-specific antibody avidity index in the ELISA was determined at months 12, 24, and 36 as previously described, and will be measured again at months 84 and 108.^13^ For the ELISA testing, the same lot of HPV16 and HPV18 VLPs, positive control, negative control, and internal reference standard were used for all DoRIS samples from month 0 to month 60, although samples were tested in different calendar years. In addition, the same positive control acceptability range was used. When testing the month 60 samples, for both HPV16 and HPV18, the laboratory retested a subset of 19 seronegative and 24 seropositive samples from earlier timepoints to evaluate drift of the assay results over time. All seronegative samples were found to be seronegative on retesting. The retest results from the seropositive samples were not significantly different from the original results for either HPV16 (p=0·34, by Wilcoxon signed rank test for matched pairs) or HPV18 (p=0·77). A description of the immunological assays at each timepoint is provided in the appendix (p 1).

### Outcomes

The primary outcomes of the main DoRIS trial related to HPV16 and HPV18 antibody responses at month 24 and have been reported previously.^13^, ^15^ The primary outcome of the extended follow-up was to show non-inferiority of immune responses to one dose of HPV vaccine compared with two doses of the same vaccine by evaluating seropositivity specific to HPV16 and HPV18 at month 60. Secondary outcomes reported here are evaluation of the stability of immune responses, comparing HPV16-specific and HPV18-specific antibody GMCs at month 60 with those at earlier timepoints within the same group; and evaluation of HPV16 and HPV18 antibody avidity at month 36, comparing one dose with two doses of the same vaccine.

### Statistical analysis

The sample size for the main DoRIS trial was based on the co-primary objectives at month 24 of showing non-inferiority of HPV16 or HPV18 seropositivity comparing one dose with two or three doses, and non-inferiority of GMCs in the immunobridging analyses.^12^ The sample size for the trial extension was not prespecified; all participants in the one-dose and two-dose groups could enrol. Retention at month 36 was more than 95%; therefore, we expected to enrol around 150 participants per group in the extension. Allowing a 10% loss to follow-up over 24 months, we expected around 135 girls per group at month 60. If the true proportion seropositive is the same in each group, with 135 girls per group, the study would have more than 90% power to show that the lower limit of the 95% CI for the difference of one dose minus two doses was above –5%, indicating that seropositivity with the one-dose schedule was not decreased by more than 5·0%.

For all immunogenicity outcomes, the primary analysis was conducted in the per-protocol population, defined as girls who received the allocated doses of vaccine within the protocol-defined window and who were HPV antibody and DNA negative at enrolment for the specific genotype under analysis. Participants who missed visits or withdrew from the trial could still be included in the per-protocol analysis, as long as they met these criteria. As a sensitivity analysis, the analyses were repeated in all participants who received at least one dose of HPV vaccine, irrespective of their baseline antibody or HPV DNA status (total vaccinated cohort).

We tabulated the number and proportion of girls who were seropositive for antibodies specific to HPV16 or HPV18 at month 60. For each vaccine type and HPV genotype, we calculated the difference (one dose minus two doses) in the proportion seropositive and the 95% CI for the difference using the exact method of Chan and Zhang.^19^ Non-inferiority of seropositivity was concluded if the lower bound of the two-sided 95% CI for the difference was above –5%.

For the evaluation of antibody GMC and avidity, we first log_10_-transformed HPV genotype-specific antibody concentrations and antibody avidity indices; those below the assay cutoff were given a value of half the cutoff before log transformation. The arithmetic mean log_10_ antibody concentration, log_10_ avidity index, and their 95% CIs were calculated for each group, assuming a normal distribution. Normality was assessed graphically using normal plots.

We assessed stability of the immune responses by estimating the fold change in HPV16 and HPV18 GMCs between month 60 and the earlier timepoints (months 36, 24, and 12). For each HPV genotype and vaccine type, we fitted a linear mixed-effects model with log_10_ antibody concentration as the response variable, dose group, timepoint, and a dose group–time interaction term as fixed effects, and participant as a random effect to account for correlation of repeated measurements within participants. The change over time in HPV16 or HPV18 log_10_ concentrations (eg, month 60 minus month 36) was estimated from this model, and the GMC ratio (month 60 over month 36) and its 95% CI were obtained by back transformation.

We compared HPV16 and HPV18 antibody avidity indices at month 36 between the one-dose and two-dose groups by calculating the difference in HPV genotype-specific log_10_ avidity index (one dose minus two doses) and its 95% CI; the geometric mean avidity index ratio and its 95% CI were obtained by back transformation.

### Role of the funding source

The funders of the study had no role in study design, data collection, data analysis, data interpretation, or writing of the report.

## Results

**Figure 1:**
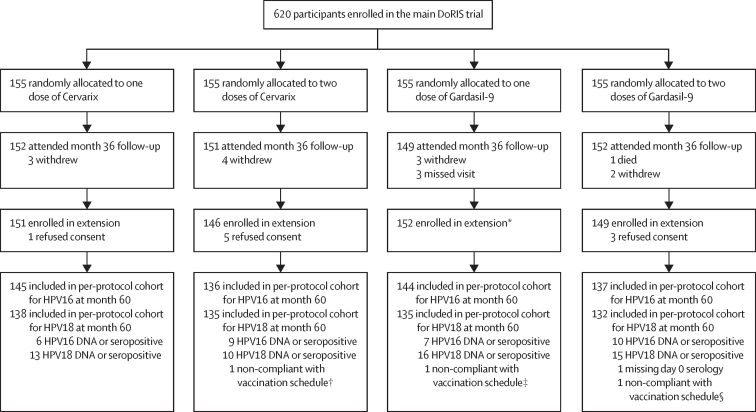
Trial profile HPV=human papillomavirus. *The three participants who missed the month 36 visit were contacted by the study team and invited to enrol in the extension; all consented to enrol and attended the month 60 visit. †Received last dose out of window (1 day early). ‡Vaccinated through the Tanzanian national programme between month 24 and month 36. §Received two-valent vaccine in error.

Of the 930 girls originally enrolled into the main DoRIS trial, 620 (67%) were randomly allocated to the one-dose and two-dose groups and were eligible to enrol in the long-term follow-up extension to the trial. Enrolment into the extension was between March 21, 2021, and Aug 31, 2022. Of the 620 eligible participants, 598 (96%) were enrolled, 13 (2%) withdrew or were lost to follow-up by month 36, and nine (1%) refused consent (figure 1). Of the 598 enrolled participants, 595 (99%) received their scheduled doses within the protocol-defined window; the three who did not were excluded from the per-protocol analyses but included in the total vaccinated cohort analyses.

Baseline characteristics at enrolment to the main trial were similar between the trial groups (table 1). One girl was positive for HPV16 and HPV18 DNA at baseline, and 32 (5%) girls were HPV16 seropositive and 55 (9%) were HPV18 seropositive at baseline. There was some evidence of a difference between groups in the proportion with any HPV DNA (p=0·02) or any high-risk HPV DNA (p=0·06) at baseline, with prevalence in the one-dose group of the nine-valent vaccine being slightly higher than in the other groups (table 1).

**Table 1:** Baseline characteristics of participants in DoRIS trial long-term follow-up

		**One dose Cervarix (N=151)**	**Two doses Cervarix (N=146)**	**One dose Gardasil-9 (N=152)**	**Two doses Gardasil-9 (N=149)**
Age, years		10 (9–12)	10 (9–12)	10 (9–12)	11 (10–13)
Age group					
	9–10 years	83 (55%)	74 (51%)	88 (58%)	66 (44%)
	11–12 years	39 (26%)	39 (27%)	39 (26%)	45 (30%)
	13–14 years	29 (19%)	33 (23%)	25 (16%)	38 (26%)
Years lived in Mwanza, Tanzania					
	Entire life	114 (75%)	116 (79%)	115 (76%)	119 (80%)
	>5 years	19 (13%)	17 (12%)	18 (12%)	17 (11%)
	≤5 years	18 (12%)	13 (9%)	19 (13%)	13 (9%)
School type					
	Primary	121 (80%)	117 (80%)	125 (82%)	117 (79%)
	Secondary	30 (20%)	29 (20%)	27 (18%)	32 (21%)
Passed menarche					
	Yes	19 (13%)	18 (12%)	17 (11%)	20 (13%)
	No	132 (87%)	128 (88%)	135 (89%)	129 (87%)
Ever cleansed vagina					
	Yes	15 (10%)	14 (10%)	14 (9%)	11 (7%)
	No	136 (90%)	132 (90%)	138 (91%)	138 (93%)
Ever had sex					
	Yes	1 (<1%)	2 (1%)	1 (<1%)	4 (3%)
	No	150 (99%)	144 (99%)	151 (99%)	145 (97%)
HPV16 DNA positive					
	Yes	0	0	1 (<1%)	0
	No	151 (100%)	146 (100%)	151 (99%)	149 (100%)
HPV18 DNA positive					
	Yes	0	0	1 (<1%)	0
	No	151 (100%)	146 (100%)	151 (99%)	149 (100%)
Any high-risk HPV genotype DNA					
	Yes	0	2 (1%)	6 (4%)	2 (1%)
	No	151 (100%)	144 (99%)	146 (96%)	147 (99%)
Any HPV genotype DNA					
	Yes	0	2 (1%)	7 (5%)	2 (1%)
	No	151 (100%)	144 (99%)	145 (95%)	147 (99%)
HPV16 seropositive					
	Yes	6 (4%)	9 (6%)	7 (5%)	10 (7%)
	No	145 (96%)	137 (94%)	145 (95%)	129 (93%)
HPV18 seropositive					
	Yes	13 (9%)	10 (7%)	16 (11%)	16 (11%)
	No	138 (91%)	136 (93%)	136 (89%)	133 (89%)

Data are median (IQR) or n (%). Characteristics were recorded at enrolment to the main DoRIS trial (ie, before vaccination) among girls in the one-dose and two-dose groups who consented to long-term follow-up.

All 598 participants attended the month 60 visit. In the per-protocol analysis, we included 289 (95%) of 303 participants in the one-dose groups and 273 (93%) of 295 in the two-dose groups in the analysis of HPV16 antibody responses, and 273 (90%) in the one-dose groups and 267 (91%) in the two-dose groups in the analysis of HPV18. In the one-dose groups, 288 (>99%) of 289 participants in the two-valent vaccine group were seropositive for HPV16 antibodies at month 60, and 261 (96%) of 273 were seropositive for HPV18 (table 2). In the two-dose groups, all 273 participants were seropositive for HPV16, and 130 (98%) of 132 in the nine-valent vaccine group were seropositive for HPV18. Non-inferiority of HPV16 antibody seropositivity at month 60 was met for one dose compared with two doses of both vaccines. Non-inferiority of HPV18 seropositivity was not met for either vaccine.

**Table 2:** Antibody seropositivity at month 60 after one or two doses of HPV vaccine (per-protocol cohort)

	**One dose**		**Two doses**		Difference in seropositivity*(one dose minus two doses)
	N	Number seropositive*	N	Number seropositive*	
**Cervarix (two-valent)**					
HPV16	145	144 (99%)	136	136 (100%)	−0·7% (−4·6 to 3·1)
HPV18	138	135 (98%)	135	135 (100%)	−2·2% (−7·1 to 1·5)
**Gardasil-9 (nine-valent)**					
HPV16	144	144 (100%)	137	137 (100%)	0
HPV18	135	126 (93%)	132	130 (98%)	−5·2% (−12·1 to 0·5)

Data are N, n (%), or difference (95% CI). Data were recorded at month 60 for participants who were ELISA antibody negative and DNA negative at baseline (before vaccination) for the HPV genotype under analysis. HPV=human papillomavirus.

* Seropositivity is defined as antibody concentrations above the laboratory-determined cutoff (1·309 IU/mL for HPV16 and 1·109 IU/mL for HPV18).

Similar results were seen at month 36, with 287 (>99%) of 288 participants in the one-dose groups being seropositive for HPV16, and non-inferiority of seropositivity comparing one dose with two doses being met for both vaccines (appendix p 2). A slightly higher proportion of participants in the one-dose groups were seropositive for HPV18 at month 36 than at month 60 (268 [99%] of 272 *vs* 261 [96%] of 273). Non-inferiority of HPV18 seropositivity comparing one dose with two doses at month 36 was met for the two-valent vaccine but not the nine-valent vaccine.

**Figure 2:**
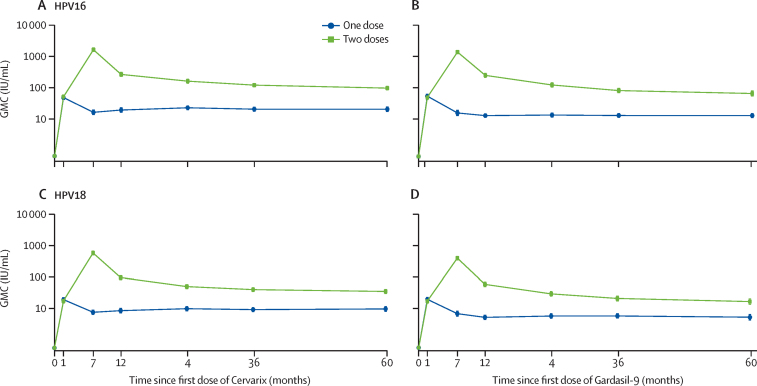
Antibody GMCs over time by number of vaccine doses and study visit (per-protocol cohort) HPV16-specific antibody GMCs with the two-valent (A) and nine-valent (B) vaccine, and HPV18-specific antibody GMCs with the two-valent (C) and nine-valent (D) vaccine. GMC=geometric mean concentration. HPV=human papillomavirus.

In the per-protocol analysis of both vaccines and HPV genotypes, antibody GMCs in the one-dose groups remained relatively constant from month 12 to month 60, with little evidence of a difference between month 60 and earlier timepoints (table 3, figure 2). In contrast, HPV16 and HPV18 antibody GMCs in the two-dose groups of both vaccines peaked at month 7 and then slowly declined thereafter. HPV16 and HPV18 antibody GMCs at month 60 in the two-dose groups, although substantially higher than in the one-dose groups, were around 20% lower than at month 36 and 65–70% lower than at month 12.

**Table 3:** Stability of GMCs from month 12 to month 60 (per-protocol cohort)

	**One dose**			**Two doses**		
	N*	GMC, IU/mL†	GMC ratio, month 60 : visit month‡	N*	GMC, IU/mL†	GMC ratio, month 60 : visit month‡
**Cervarix HPV16**						
Month 12	147	19·4 (16·6–22·7)	1·06 (0·96–1·17)	140	267·6 (231·9–308·8)	0·36 (0·33–0·40)
Month 24	148	22·9 (19·9–26·4)	0·90 (0·82–0·99)	141	162·7 (141·1–187·7)	0·59 (0·54–0·66)
Month 36	146	20·7 (17·9–23·9)	1·00 (0·90–1·10)	141	121·5 (107·4–137·4)	0·80 (0·72–0·88)
Month 60	145	20·5 (17·3–24·3)	**..**	136	97·6 (85·8–111·0)	**..**
**Cervarix HPV18**						
Month 12	140	8·6 (7·3–10·0)	1·14 (1·02–1·26)	139	96·0 (83·1–110·9)	0·36 (0·33–0·40)
Month 24	141	9·9 (8·5–11·5)	0·98 (0·89–1·09)	140	50·0 (43·4–57·8)	0·70 (0·63–0·77)
Month 36	139	9·3 (8·0–10·7)	1·05 (0·95–1·17)	140	40·1 (34·9–46·1)	0·87 (0·78–0·96)
Month 60	138	9·7 (8·2–11·6)	**..**	135	35·1 (30·4–40·4)	**..**
**Gardasil-9 HPV16**						
Month 12	145	13·2 (11·5–15·0)	1·00 (0·92–1·09)	142	252·8 (219·2–291·5)	0·27 (0·24–0·29)
Month 24	145	13·7 (11·9–15·8)	0·96 (0·88–1·04)	141	124·9 (107·2–145·5)	0·54 (0·49–0·59)
Month 36	142	13·2 (11·6–15·1)	0·99 (0·91–1·08)	140	82·7 (70·7–96·8)	0·82 (0·75–0·89)
Month 60	144	13·1 (11·3–15·3)	**..**	137	66·8 (55·9–79·7)	**..**
**Gardasil-9 HPV18**						
Month 12	136	5·2 (4·5–6·1)	1·01 (0·92–1·11)	137	58·8 (50·2–68·9)	0·29 (0·26–0·32)
Month 24	136	5·7 (4·9–6·8)	0·92 (0·84–1·01)	136	29·3 (24·7–34·7)	0·57 (0·52–0·63)
Month 36	133	5·8 (4·9–6·7)	0·91 (0·83–1·00)	135	20·9 (17·6–24·9)	0·80 (0·73–0·88)
Month 60	135	5·3 (4·3–6·4)	**..**	132	16·6 (13·6–20·3)	**..**

Data are N, GMC (95% CI), or GMC ratio (95% CI). GMC=geometric mean concentration. HPV=human papillomavirus.

* Population is DoRIS participants who were ELISA antibody negative and DNA negative at baseline (before vaccination) for the HPV genotype under analysis.

† ELISA serum antibody GMC.

‡ Estimated with linear mixed-effects model with log antibody concentration as the response and dose group, timepoint, and a dose group–time interaction term as fixed effects, and participant as a random effect to account for correlation of repeated measurements within participants.

In the per-protocol analysis, there was no evidence of a difference between the one-dose and two-dose groups in HPV16 and HPV18 geometric mean antibody avidity index at month 36 for either vaccine (table 4, appendix p 8). Geometric mean avidity index ratios were around 1·0, with the lower limit of the 95% CI above 0·90 for all comparisons. Immunogenicity results among the total vaccinated cohort were similar to those in the per-protocol analysis for both vaccines and both HPV genotypes (appendix pp 3–5, 9).

**Table 4:** GMAI at month 36 after one or two doses of HPV vaccine (per-protocol cohort)

	**Recipients of one dose**	**GMAI for one dose**	**Recipients of two doses**	**GMAI for two doses**	**Geometric mean avidity index ratio, one dose : two doses**
**Cervarix**					
HPV16	146	2·99 (2·91–3·09)	141	3·06 (3·01–3·12)	0·98 (0·95–1·01)
HPV18	139	1·80 (1·74–1·87)	140	1·83 (1·78–1·88)	0·98 (0·93–1·02)
**Gardasil-9**					
HPV16	142	2·92 (2·84–3·01)	140	2·98 (2·92–3·04)	0·98 (0·95–1·01)
HPV18	133	2·04 (1·98–2·11)	135	2·08 (2·03–2·13)	0·98 (0·94–1·02)

Data are n, mean (95% CI), or ratio (95% CI). DoRIS participants who were ELISA antibody negative and DNA negative at baseline (pre-vaccination) for the HPV genotype under analysis. GMAI=geometric mean antibody avidity index. HPV=human papillomavirus.

There were 40 serious adverse events experienced by 31 (5%) of the 620 girls originally enrolled in the one-dose and two-dose groups (including 22 who were not enrolled in the extension) up to month 60 (appendix pp 6–7). Hospitalisation for malaria was the most common serious adverse event (34 events in 25 girls). A girl aged 10 years in the two-dose nine-valent vaccine group, who had received her first dose 4 months previously, died from severe malaria. There was no evidence of a difference in the number of serious adverse events between groups, and no serious adverse event was considered to be related to the vaccine.

## Discussion

This is the first randomised trial to assess the long-term durability of antibody responses after a single dose in girls in the primary target age for HPV vaccination. This is also the first study of long-term immunogenicity of a single dose of nine-valent vaccine in any age group. We showed that HPV16 and HPV18 immune responses plateau at around 12 months and remain relatively constant to 5 years after a single dose of either the two-valent or nine-valent HPV vaccine. HPV16 seropositivity rates at 5 years after a single dose were non-inferior to those after two doses for both vaccines. Although we did not show non-inferiority of HPV18 seropositivity, 98% of participants in the one-dose two-valent vaccine group and 93% in the one-dose nine-valent vaccine group were HPV18 seropositive at 5 years. HPV16 and HPV18 antibody avidity at 3 years after vaccination did not differ between the one-dose and two-dose groups for either vaccine.

Our results are consistent with findings in older females in the CVT and IARC India studies, where antibody concentrations after a single dose of the two-valent vaccine or the four-valent vaccine followed similar trajectories over time and have been stable for a decade.^9^, ^14^ In the CVT, 97% of single-dose recipients were seropositive for HPV16 and 93% were seropositive for HPV18 at 11 years. In the IARC India trial, HPV16 seropositivity among single-dose recipients at 10 years was 96% and HPV18 seropositivity was 97%. Both studies have shown long-term (up to 11 years) single-dose efficacy against persistent HPV16 or HPV18 infection.

Our findings—that HPV16 and HPV18 antibody avidity in the one-dose groups was similar to that after two doses—are similar to a study in the Netherlands of girls who were vaccinated with the two-valent vaccine at the age of 12 years through the national programme.^20^ In that study, HPV16 antibody avidity at 5 years did not differ between those who received only a single dose and those who received two doses, and HPV18 antibody avidity was higher in the single-dose recipients. In the CVT, where women were vaccinated at age 18–25 years, HPV16 antibody avidity was 5–10% lower among women who received one dose than those who received three doses, but was relatively constant over time to 11 years.^21^ Avidity is believed to reflect the quality of antibodies after vaccination following affinity maturation and antigen-driven B-cell selection. Our results, and those of the Netherlands and CVT studies, suggest that a single dose of VLP HPV vaccine, irrespective of its adjuvant, can generate robust and stable immune responses through B-cell activation. Several biological mechanisms by which a single dose can produce stable antibody responses have been proposed, linked to the repetitive structure and spacing of the VLP epitopes, which trigger a particularly effective cascade of immune responses.^22^

As we previously observed at month 24, HPV16 and HPV18 antibody GMCs at month 60 were higher with the two-valent vaccine than with the nine-valent vaccine. These findings are similar to those in the KEN SHE trial, which compared the same two HPV vaccines as the DoRIS trial, and to other studies that compared the two-valent and four-valent vaccines.^16^, ^23^, ^24^ Despite the differences in antibody GMCs, the vaccines have similar extremely high efficacy against persistent HPV16 or HPV18 infection. In addition, as we also observed at month 24, HPV18 antibody GMCs and seropositivity at month 60 were lower than those for HPV16 for both vaccines. These results were not unexpected and have been reported in other studies. A trial of three doses of the nine-valent versus four-valent vaccines among women aged 16–26 years found that 18% in the nine-valent group and 23% in the four-valent group no longer had detectable HPV18 antibodies at 3·5 years.^25^ Similarly, an extended follow-up of women who received three doses of the four-valent vaccine found that 35% were no longer HPV18 seropositive at 5 years, despite sustained efficacy against HPV18 infection.^26^ The mechanism for protection among women who become HPV18 seronegative several years after vaccination is still unclear. It is uncertain whether a minimum serum antibody concentration must be maintained for protection, or whether exposure to the virus can activate memory B cells to produce neutralising antibody locally in the genital tract. The results from these efficacy studies suggest that some of the vaccine protection is likely to be mediated through immune memory.

Combined with our immunobridging results, where immune responses at 2 years after a single dose were non-inferior to those in the KEN SHE, CVT, and IARC India studies where efficacy against persistent HPV infection had been shown, our long-term immunogenicity results at 5 years suggest that a single dose of the vaccine given to girls aged 9–14 years is likely to protect against HPV16 or HPV18 infection once girls pass sexual debut. Ongoing data from the CVT and IARC India studies also confirm that, in those observational cohorts, efficacy in the single-dose recipients is sustained for up to a decade.

Study strengths include excellent retention at 5 years after vaccination, and enrolment of girls from a malaria-endemic setting in a country that has a very high prevalence and incidence of HPV infection and high rates of cervical cancer.^27^, ^28^, ^29^ Our results are, therefore, generalisable to other high-burden countries. The immunological assays for this study were conducted in the same laboratory that evaluated earlier immune responses from the DoRIS trial, using the same lot of ELISA VLPs, standards, and critical reagents, with retesting of samples from earlier timepoints, to minimise potential assay variability. The laboratory is also performing HPV immunological assays for other studies of the single-dose schedule, allowing comparison of results across these studies.^30^ The inclusion of HPV16 and HPV18 antibody avidity is important for confirming the robustness of the immune response to a single dose.

Our study has several limitations. Our sample size does not allow us to directly evaluate efficacy in the DoRIS trial, although we have bridged our immune response to those in studies with efficacy results. We did not show non-inferiority of HPV18 seropositivity when comparing one dose with two doses, although more than 93% of girls in the one-dose groups remained HPV18 seropositive and the clinical significance of the loss of seropositivity is unclear. Our data on durability are based on 5 years of follow-up; however, based on our other studies in Mwanza, the median age of sexual debut among females in this region is around 17 years.^29^, ^31^ We are continuing follow-up of participants to 9 years after vaccination, which will provide further information on the durability of single-dose immune responses in this age group at a timepoint where most participants are likely to have passed sexual debut.

In conclusion, a single dose of the two-valent or nine-valent vaccine in healthy African girls living in a malaria-endemic region and who are in the primary target age for vaccination continues to result in sustained, stable antibody responses 5 years after vaccination. Antibody kinetics are similar to those observed in other studies in older females from different geographies in whom efficacy has been shown. These data, combined with recent efficacy data on single-dose regimens from the KEN SHE randomised controlled trial and long-term follow-up data from India and Costa Rica, continue to support the recent WHO recommendation for a single-dose HPV vaccine regimen.^17^ The potential savings and vaccine supplies that might become available if single-dose vaccination programmes are introduced could permit catch-up vaccination for those who failed to be vaccinated in previous years and could allow vaccination of multi-age cohorts, at least temporarily. Ongoing surveillance for potential waning immunity following a single dose is important and follow-up of the existing single-dose study cohorts, including DoRIS participants, is underway. Encouragingly, as of March, 2024, 38 countries have introduced HPV vaccination using a single-dose regimen or made a recommendation to switch their current HPV vaccination programmes to a single-dose HPV vaccination strategy.^32^ Our data might reassure countries considering a potential HPV vaccination programme with a single-dose strategy.

### Contributors

### Data sharing

De-identified participant data presented in this manuscript can be made available after publication following written request to the London School of Hygiene & Tropical Medicine and the Mwanza Intervention Trials Unit (MITU), Tanzania. Requests must be accompanied by an analysis plan, which will be reviewed by the MITU Data Sharing Committee and lead investigators for each trial. Requesting researchers will be required to sign a Data Access Agreement if approval is given.

## Declaration of interests

DW-J reports grants from the Bill & Melinda Gates Foundation and GSK Biologicals. KB reports grants from the Bill & Melinda Gates Foundation and Merck. All other authors declare no competing interests.
