# Supplementary material for: Durability of immunogenicity at 5 years after a single dose of human papillomavirus vaccine compared with two doses in Tanzanian girls aged 9–14 years: results of the long-term extension of the DoRIS randomised trial
Source: Lancet Glob Health. 2025 Jan 29;13(2):e319–28. doi: 10.1016/S2214-109X(24)00477-7 (PMC11783036; doi:10.1016/S2214-109X(24)00477-7)

THE LANCET
Global Health

Supplementary appendix

This appendix formed part of the original submission and has been peer reviewed. We post it as supplied by the authors.

Supplement to: Watson-Jones D, Changalucha J, Maxwell C, et al. Durability of immunogenicity at 5 years after a single dose of human papillomavirus vaccine compared with two doses in Tanzanian girls aged 9–14 years: results of the long-term extension of the DoRIS randomised trial. *Lancet Glob Health* 2025; **13:** e319–28.

Supplementary material

Table of contents

Supplementary Table 1. Laboratory assays used in DoRIS trial 1

Supplementary Table 2. Comparisons of antibody seropositivity at M36 post HPV vaccination with 1 or 2 doses of HPV vaccine in DoRIS trial (per protocol cohort1) 2

Supplementary Table 3. Comparisons of antibody seropositivity at M36 and M60 post HPV vaccination with 1 or 2 doses of HPV vaccine in DoRIS trial (total vaccinated cohort[1]) 3

Supplementary Table 4. Stability of geometric mean concentrations (GMC) between M12 to M60 in DoRIS trial (total vaccinated cohort[1]) 4

Supplementary Table 5. Number of participants with at least one serious adverse event, and number of events, by trial arm from enrolment to Month 60 (total vaccinated cohort) 6

Supplementary Table 6. Serious adverse event by diagnosis and trial arm, from enrolment to Month 60 visit (total vaccinated cohort) 7

Supplementary Figure 1. Distribution of HPV-16 and HPV-18 antibody avidity index (AI) at 36 months by arm 8

Supplementary Figure 2. Distribution of HPV-16 and HPV-18 antibody concentrations (IU/mL) at 60 months by arm (total vaccinated cohort) 9

Supplementary Table 1. Laboratory assays used in DoRIS trial

Assay	Marker	Timepoint	Sample type	Laboratory
L1 VLP ELISA	HPV-16/18 IgG antibody concentrations	M0, 1, 7, 12, 24, 36, 60, 84 & 108	Serum	Frederick National Laboratory for Cancer Research, USA
ELISA-based avidity	HPV 16/18 IgG antibody avidity index	M0, 12, 24 & 36, 84 & 108	Serum	Frederick National Laboratory for Cancer Research, USA
HPV Multiplex immunoassay	HPV-6, 11, 16, 18, 31, 33, 45, 52 & 58 IgG antibody concentrations	M0, 12, 24, 36, 60, 84 & 108	Serum	Frederick National Laboratory for Cancer Research, USA
Pseudovirion (PsV) Luminex	HPV-6, 11, 16, 18, 31, 33, 45, 52 & 58 IgG antibody concentrations	M0, 1, 7, 12, 24, 36, 60, 84 & 108	Serum	Karolinska Institute, Sweden
Memory B cell ELISPOT	HPV 16/18-specific memory B cell response	M0, 1, 7, 12, 24 & 36	PBMC	Centre for Immunology and Infection, York, UK
qPCR[1]	Malaria	M0, 1, 2 & 6	Dried blood spot	LSHTM, UK
Roche linear array	HPV DNA	M0	Vaginal swab	Catalan Institute of Oncology, Barcelona, Spain
HIV rapid test[2]	HIV serostatus	M0	Serum	NIMR Mwanza, Tanzania

[1]Quantitative PCR. [2]Two rapid tests (serial testing), with second test done only if first test is reactive; rapid tests repeated if discordant. Participants with persistently discordant results tested by ELISA.

Supplementary Table 2. Comparisons of antibody seropositivity at M36 post HPV vaccination with 1 or 2 doses of HPV vaccine in DoRIS trial (per protocol cohort1)

	1 dose		2 doses		Difference in seropositivity[2] (exact 95% CI)
	**N**	**Seropositive[2] (%)**	**N**	**Seropositive[2] (%)**	**1 dose – 2 dose**
**Month 36**					
**Cervarix®**					
HPV-16	146	145 (99.3%)	141	141 (100.0%)	-0.7% (-3.9, 2.0)
HPV-18	139	137 (98.6%)	140	139 (99.3%)	-0.7% (-4.6, 2.7)
**Gardasil-9®**					
HPV-16	142	142 (100.0%)	140	140 (100.0%)	0
HPV-18	133	131 (98.5%)	135	135 (100.0%)	-1.5% (-5.5, 1.3)

[1]DoRIS participants who were ELISA antibody negative and DNA negative at baseline (pre-vaccination) for the HPV genotype under analysis. [2]Seropositivity defined as antibody concentrations above the laboratory determined cut-off (HPV16 = 1.309 IU/mL; HPV18 = 1.109 IU/mL).

Supplementary Table 3. Comparisons of antibody seropositivity at M36 and M60 post HPV vaccination with 1 or 2 doses of HPV vaccine in DoRIS trial (total vaccinated cohort[1])

	1 dose			2 doses			Difference in seroconversion[3] (exact 95% CI)
	N	Seropositive[2] (%)	Seroconverted[3] (%)	N	Seropositive[2] (%)	Seroconverted[3] (%)	1 dose – 2 dose
**Cervarix®**							
**Month 36**							
HPV16	152	151 (99.3%)	145 (95.4%)	151	151 (100.0%)	142 (94.0%)	1.4% (-4.1, 7.0)
HPV18	152	150 (98.7%)	137 (90.1%)	151	150 (99.3%)	140 (92.7%)	-2.6% (-9.3- 4.0)
**Month 60**							
HPV16	151	150 (99.3%)	144 (95.4%)	146	146 (100.0%)	137 (93.8%)	-0.7% (-4.4- 2.7)
HPV18	151	148 (98.0%)	135 (89.4%)	146	146 (100.0%)	136 (93.2%)	-2.0% (-6.5, 1.5)
**Gardasil-9®**							
**Month 36**							
HPV16	149	149 (100.0%)	143 (96.0%)	152	152 (100.0%)	141 (92.8%)	3.2% (-2.4, 9.0)
HPV18	149	147 (98.7%)	132 (88.6%)	152	152 (100.0%)	135 (88.8%)	-0.4% (-8.0, 7.1)
**Month 60**							
HPV16	152	152 (100.0%)	145 (95.4%)	149	149 (100.0%)	138 (92.6%)	0
HPV18	152	143 (94.1%)	127 (83.6%)	149	147 (98.7%)	130 (87.2%)	-4.6% (-10.7, 0.4)

[1]DoRIS participants who received at least one dose of vaccine, irrespective of their HPV DNA or serostatus at baseline. [2]Antibody concentrations above the laboratory determined cut-off (HPV16 = 1.309 IU/mL; HPV18 = 1.109 IU/mL). [3]Seroconversion defined as antibody concentrations above the laboratory determined cut-off at the relevant visit, among girls who were seronegative at baseline

Supplementary Table 4. Stability of geometric mean concentrations (GMC) between M12 to M60 in DoRIS trial (total vaccinated cohort[1])

	1 dose		2 doses	
	N[1]	GMC[2] (95% CI) (IU/mL)	N[1]	GMC[2] (95% CI) (IU/mL)
**Cervarix®**				
**HPV 16**				
Month 12	153	19.5 (16.7, 22.7)	150	267.2 (232.9, 306.7)
Month 24	154	22.7 (19.8, 26.1)	151	162.8 (142.1, 186.5)
Month 36	152	20.6 (17.9, 23.7)	151	121.7 (108.2, 136.9)
Month 60	151	20.5 (17.4, 24.1)	146	96.0 (85.0, 108.5)
GMC ratio[3] (M60 /M12) (95% CI)		*1.06 (0.96, 1.16)*		*0.36 (0.32, 0.39)*
GMC ratio[3] (M60 /M24) (95% CI)		*0.91 (0.83, 1.00)*		*0.58 (0.53, 0.64)*
GMC ratio[3] (M60 /M36) (95% CI)		*1.00 (0.91, 1.10)*		*0.78 (0.71, 0.86)*
**HPV 18**				
Month 12	153	8.3 (7.1, 9.6)	150	92.7 (80.0, 107.3)
Month 24	154	9.6 (8.3, 11.1)	151	50.8 (44.2, 58.3)
Month 36	152	9.0 (7.8, 10.4)	151	40.4 (35.3, 46.2)
Month 60	151	9.5 (8.0, 11.2)	146	35.0 (30.5, 40.1)
GMC ratio[3] (M60 /M12) (95% CI)		*1.14 (1.03, 1.26)*		*0.38 (0.34, 0.42)*
GMC ratio[3] (M60 /M24) (95% CI)		*0.99 (0.89, 1.09)*		*0.69 (0.62, 0.76)*
GMC ratio[3] (M60 /M36) (95% CI)		*1.05 (0.95, 1.17)*		*0.86 (0.78, 0.96)*
**Gardasil-9®**				
**HPV 16**				
Month 12	152	13.5 (11.8, 15.5)	154	248.8 (217.3, 284.8)
Month 24	152	14.1 (12.2, 16.3)	153	122.8 (106.2, 142.0)
Month 36	149	14.0 (12.1, 16.2)	152	82.5 (71.0, 95.8)
Month 60	152	13.8 (11.8, 16.1)	149	66.0 (55.9, 78.0)
GMC ratio[3] (M60 /M12) (95% CI)		*1.02 (0.93, 1.11)*		*0.27 (0.25, 0.29)*
GMC ratio[3] (M60 /M24) (95% CI)		*0.98 (0.90, 1.07)*		*0.54 (0.50, 0.59)*
GMC ratio[3] (M60 /M36) (95% CI)		*0.99 (0.90, 1.08)*		*0.81 (0.74, 0.88)*
**HPV 18**				
Month 12	152	5.4 (4.7, 6.3)	154	58.2 (50.1, 67.7)
Month 24	152	6.0 (5.2, 7.0)	153	29.0 (24.7, 34.0)
Month 36	149	6.1 (5.3, 7.1)	152	20.9 (17.8, 24.6)
Month 60	152	5.7 (4.7, 6.8)	149	16.7 (13.8, 20.3)
GMC ratio[3] (M60 /M12) (95% CI)		*1.04 (0.95, 1.14)*		*0.29 (0.27, 0.32)*

GMC ratio[3] (M60 /M24) (95% CI)	*0.94 (0.86, 1.03)*	*0.58 (0.53, 0.64)*
GMC ratio[3] (M60 /M36) (95% CI)	*0.92 (0.84, 1.01)*	*0.81 (0.74, 0.89)*

[1]DoRIS participants who received at least one dose of vaccine, irrespective of their HPV DNA or serostatus at baseline. [2]ELISA serum antibody geometric mean concentrations (GMC). [3]Estimated with linear mixed effects model with log antibody concentration as the response and dose group, time point, and a dose group-time interaction term as fixed effects, and participant as a random effect to account for correlation of repeated measurements within participants.

Supplementary Table 5. Number of participants with at least one serious adverse event, and number of events, by trial arm from enrolment to Month 60 (total vaccinated cohort)

		1 dose Cervarix® (N=155)	2 doses Cervarix® (N=155)	1 dose Gardasil-9® (N=155)	2 doses Gardasil-9® (N=155)	Total (N=620)[1]
**All SAEs**	Number of girls (%)	10 (6.5 %)	4 (2.6 %)	8 (5.2 %)	9 (5.8 %)	31 (5.0 %)
	(Number of events)	*(17)*	*(4)*	*(8)*	*(11)*	*(40)*
**Components of SAEs**						
Death	Number of girls (%)	0 (-)	0 (-)	0 (-)	1 (0.6 %)	1 (0.2 %)
	(Number of events)	*(0)*	*(0)*	*(0)*	*(1)*	*(1)*
Hospitalisation	Number of girls (%)	10 (6.5 %)	3 (1.9 %)	7 (4.5 %)	8 (5.2 %)	28 (4.5 %)
	(Number of events)	(17)	(3)	(7)	(10)	(37)
Life-threatening condition	Number of girls (%)	0 (-)	0 (-)	0 (-)	0 (-)	0 (-)
Persistent disability	Number of girls (%)	0 (-)	0 (-)	0 (-)	0 (-)	0 (-)
Congenital abnormality	Number of girls (%)	0 (-)	1 (0.6 %)	0 (-)	0 (-)	1 (0.2 %)
	(Number of events)	*(0)*	*(1)*	*(0)*	*(0)*	*(1)*
Other medically important event	Number of girls (%)<br>*(Number of events)*	0 (-)<br>*(0)*	0 (-)<br>*(0)*	1 (0.6 %)<br>*(1)*	0 (-)<br>*(0)*	1 (0.2 %)<br>*(1)*

[1]Includes 22 participants who were not enrolled in the long-term follow-up extension (13 participants who were lost to follow-up/withdrawn by M36 and 9 who did not consent)

Supplementary Table 6. Serious adverse event by diagnosis and trial arm, from enrolment to Month 60 visit (total vaccinated cohort)

**Number of events**	**1 dose Cervarix®**	**2 doses Cervarix®**	**1 dose Gardasil-9®**	**2 doses Gardasil-9®**	**Total**
Severe malaria	15	3	6	11	35
Urinary tract infection	0	0	0	0	0
Gastroenteritis	0	0	1	0	1
Amoebiasis	0	0	0	0	0
Peptic ulcer disease	1	0	0	0	1
Dehydration due to fever	1	0	0	0	1
Anaemia	0	0	0	0	0
Vasovagal syncope	0	0	0	0	0
Snake bite	0	0	0	0	0
Spontaneous abortion	0	0	1	0	1
Caesarean section	0	0	0	0	0
Congenital anomaly	0	1	0	0	1
**Total events**	**17**	**4**	**8**	**11**	**40**

Supplementary Figure 1. Distribution of HPV-16 and HPV-18 antibody avidity index (AI) at 36 months by arm. Each data point represents a single individual and the line through the data points represents the median AI

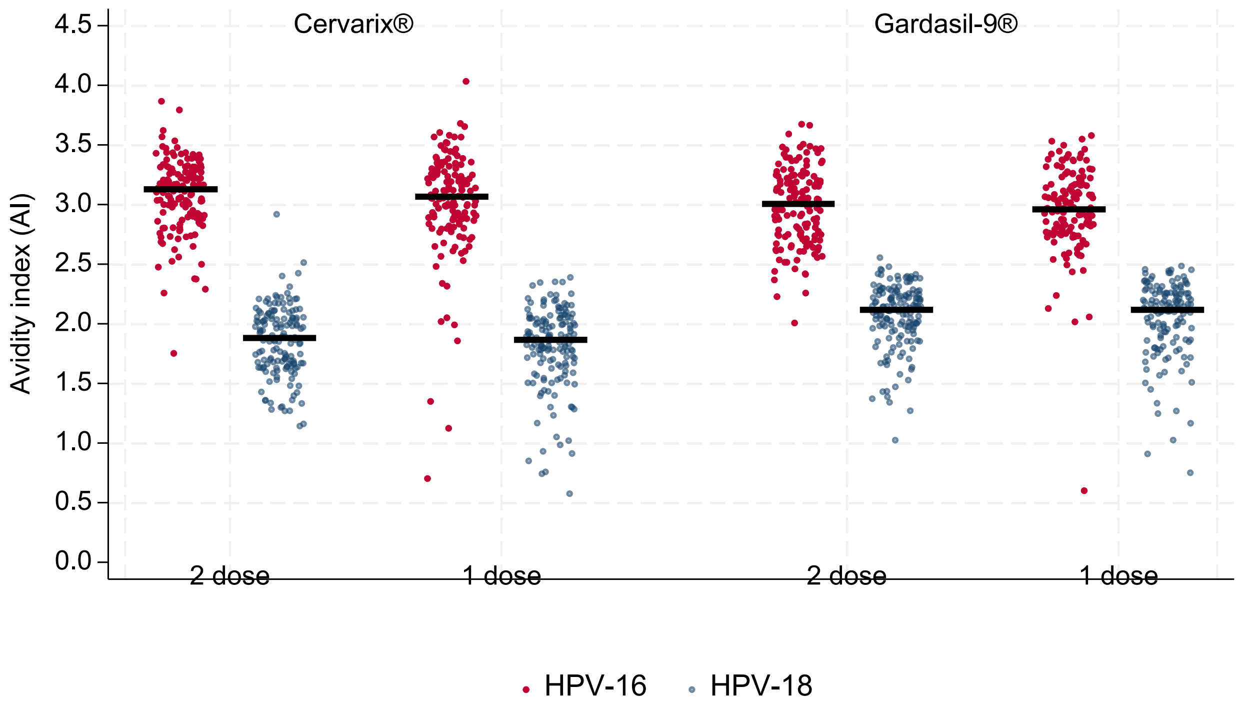

Supplementary Figure 2. Distribution of HPV-16 and HPV-18 antibody concentrations (IU/mL) at 60 months by arm (total vaccinated cohort). Each data point represents a single individual and the line through the data points represents the median concentration

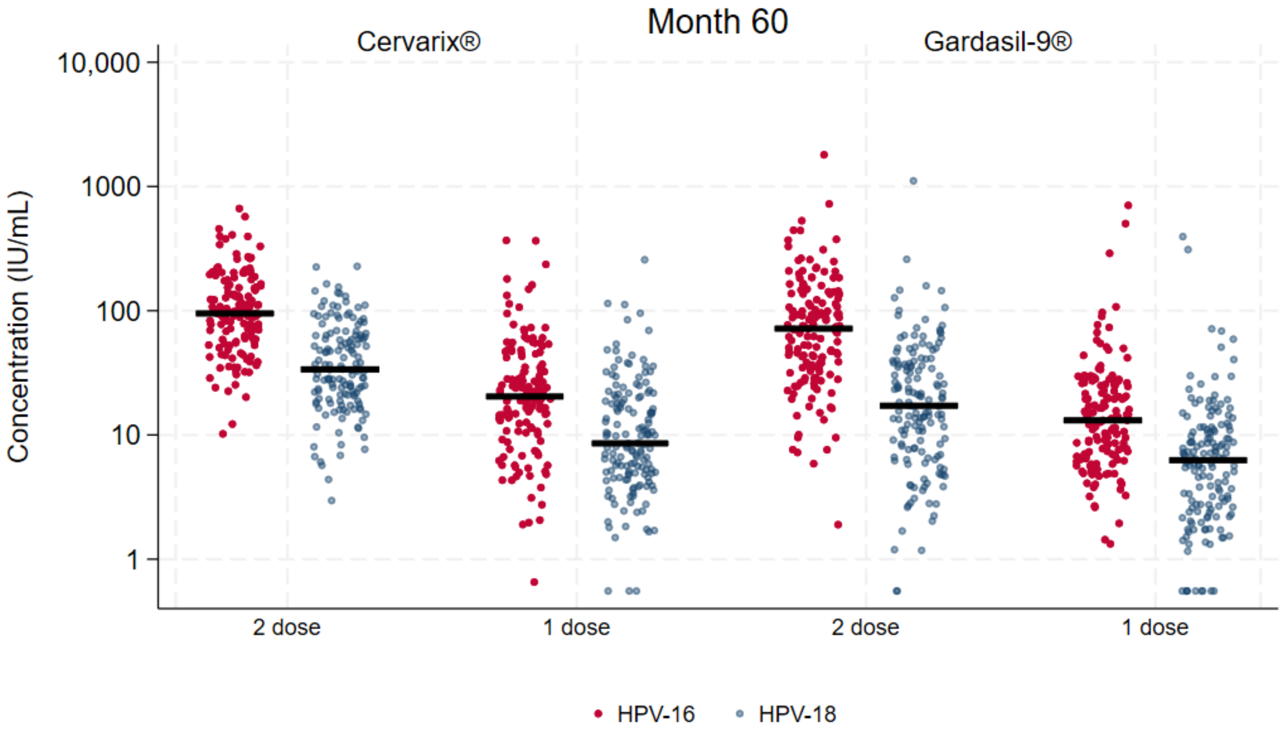

Supplement: Supplementary appendix [file mmc1.pdf]
